# Quantifying the Sensitivity of Soil Microbial Communities to Silver Sulfide Nanoparticles Using Metagenome Sequencing

**DOI:** 10.1371/journal.pone.0161979

**Published:** 2016-08-30

**Authors:** Casey L. Doolette, Vadakattu V. S. R. Gupta, Yang Lu, Justin L. Payne, Damien J. Batstone, Jason K. Kirby, Divina A. Navarro, Mike J. McLaughlin

**Affiliations:** 1 School of Agriculture Food and Wine, The University of Adelaide, Adelaide, Australia; 2 CSIRO Agriculture, Functional Microbial Ecology, Adelaide, Australia; 3 Advanced Water Management Centre (AWMC), The University of Queensland, St. Lucia, Australia; 4 School of Natural and Built Environments, University of South Australia, Adelaide, Australia; 5 CSIRO Land and Water, Environmental Contaminant Mitigation and Technologies Research Program, Adelaide, Australia; VIT University, INDIA

## Abstract

Soils are a sink for sulfidised-silver nanoparticles (Ag_2_S-NPs), yet there are limited ecotoxicity data for their effects on microbial communities. Conventional toxicity tests typically target a single test species or function, which does not reflect the broader community response. Using a combination of quantitative PCR, 16S rRNA amplicon sequencing and species sensitivity distribution (SSD) methods, we have developed a new approach to calculate silver-based NP toxicity thresholds (HCx, hazardous concentrations) that are protective of specific members (operational taxonomic units, OTUs) of the soil microbial community. At the HC20 (80% of species protected), soil OTUs were significantly less sensitive to Ag_2_S-NPs compared to AgNPs and Ag^+^ (5.9, 1.4 and 1.4 mg Ag kg^-1^, respectively). However at more conservative HC values, there were no significant differences. These trends in OTU responses matched with those seen in a specific microbial function (rate of nitrification) and *amo*A-bacteria gene abundance. This study provides a novel molecular-based framework for quantifying the effect of a toxicant on whole soil microbial communities while still determining sensitive genera/species. Methods and results described here provide a benchmark for microbial community ecotoxicological studies and we recommend that future revisions of Soil Quality Guidelines for AgNPs and other such toxicants consider this approach.

## Introduction

Silver has broad-spectrum antimicrobial properties and silver nanoparticles (AgNPs) and soluble Ag (Ag^+^) have both been demonstrated to have deleterious effects on the soil microbial biomass [1]. This in turn could lead to significant environmental and economic costs. Silver nanoparticles or Ag^+^ can be released into wastewater from AgNP-containing goods (e.g. washing machines [2]) or when AgNP-containing products are washed (e.g. textiles [3]). During wastewater treatment, the majority of AgNPs and Ag^+^ are transformed to Ag-sulfide NPs (Ag_2_S-NPs) which then adsorb to, or are incorporated in, the biosolid products of the treatment process [4]. In many countries, these biosolids are applied to land as an agricultural amendment to improve soil fertility. As a result, soil may effectively act as a sink for Ag_2_S-NPs. The use of Ag_2_S-NP–bearing biosolids as an agricultural amendment is potentially at odds with the importance of maintaining healthy soil microbial communities.

While the effects of environmental exposure of AgNPs to aboveground vegetation and specific soil fauna have been considered, the effects on soil microorganisms and biological processes are not well studied. Similarly, for Ag_2_S-NPs, only a limited number of studies have investigated the effects on terrestrial organisms. The plant biomass of wheat (*Triticum aesticum* L.) and cowpea (*Vigna unguiculata* L. Walp) decreased following a two week exposure to Ag_2_S-NPs at 6 mg Ag L^-1^ [5]. At lower Ag concentrations (1.3 mg kg^-1^), the biomass of lettuce (*Lactuca sativa*) was not affected by Ag_2_S-NPs [6] but there was evidence of effects at higher doses. In addition, Ag_2_S-NPs (at 10 mg Ag L^-1^) have also been shown to increase the mortality of the model soil organism, *Caenorhabditis elegans*, by 20% [7]. As a potential positive, in these experiments, Ag_2_S-NPs were shown to be less toxic than pristine AgNPs and Ag^+^. However, it has been shown that in aquatic environments, the potential exists for Ag_2_S-NPs to act as a slow-release form of toxic Ag^+^ [8]. Overall, this lack of information prevents the development of threshold values to quantify the risks of Ag-based NPs to soil microorganisms and hence, the ability to develop regulatory Soil Quality Guidelines (SQGs) to protect soil ecosystems.

Microbial communities are important and sensitive targets for determining the environmental hazards of manufactured NPs [9]. Microorganisms are capable of transforming metals in soil (e.g. by oxidation and methylation), but are also susceptible to toxicity from metal NPs at the cellular, community and ecosystem scales [9]. Silver NPs in particular have been shown to affect soil microbial biomass, diversity and structure. Previous studies on the effects of AgNPs on soil microorganisms have primarily focused on three main areas: 1) composition of the microbial community [10]; 2) changes in soil microbial biomass [11]; and 3) the toxicity to individual cultured microbial species [12–14]. Using ordination methods, Colman et al. (2013) showed that composition of the bacterial community in AgNP spiked soil (0.14 mg kg^-1^) was significantly different to the control 1 d after spiking (p<0.0117). However, after 50 d, there were no significant differences between treatments. At higher exposure concentrations, AgNPs have been shown to alter soil bacterial community structure. For example, Kumar et al. (2014) observed a 370-fold decrease in the number of sequence reads attributed to the genus *Rhizobium* in an arctic soil exposed to AgNPs at 660 mg kg^-1^ [15]. And following a four-month exposure to AgNPs, soil microbial biomass has been shown to decrease by 14% and 35% (3.2 and 320 μg Ag kg^-1^, respectively) [11]. However, using single species or single function approaches such as these provides a narrow and potentially unrealistic assessment of the risks of a toxicant as little understanding is gained on the broad effects to the natural soil microbial community [16].

Whole community nucleic acid sequencing can provide a powerful tool to determine the effects of Ag_2_S-NPs on total soil microbial communities. This can help analyse the effect of a stressor or toxicant *via* stress-induced changes in gene expression and can be applied to individual organisms or a whole community. For example, Roh et al. [17] investigated the effect of AgNPs on the earthworm *C*. *elegans* and found that changes to measured endpoints (e.g. reproduction) may be related to expression of the *sod-3* and *daf-12*genes [17]. Genomics tools can also be used to investigate the changes in community dynamics when exposed to a stressor. Using microbial metagenomic amplicon sequencing (TEFAP), Shah et al. [10] demonstrated a significant decrease in soil bacterial richness after a 120 d exposure to AgNPs (0.0625 mg AgNPs kg^-1^)

In the current study, instead of focusing on changes in community composition, a more detailed approach was applied to determine the toxicity of Ag_2_S-NPs to individual members within a community. This approach enables the determination of targeted toxicity thresholds for specific microbial families and genera. Toxicity thresholds are essential for setting regulatory limits for toxicants in the environment, and this approach reduces the influence of non-sensitive organisms. Specifically, a soil nitrification experiment was first carried out to determine the concentration range over which Ag^+^, AgNPs and Ag_2_S-NPs would affect a soil microbial process (nitrification). Nitrification is particularly sensitive to metal contamination and as a result is often used to assess the potential risk of metal contamination of soils [18]. A second experiment used Illumina sequencing-based analysis of the 16S rRNA amplicon to construct dose-response curves for individual soil microbial populations exposed to Ag^+^, AgNPs and Ag_2_S-NPs. Finally, toxicity endpoints (EC_20_) determined from the dose-response curves were used to construct species sensitivity distributions (SSDs); enabling comparisons to be made between the sensitivities of soil microorganisms to different Ag treatments. A SSD is a cumulative statistical distribution of toxicity values (e.g. EC_20_) for multiple species. The SSD methodology is commonly used to set water and soil quality guidelines [19–21]. This multi-disciplinary study develops a new approach to assess the sensitivity of the soil microbial community to Ag^+^, AgNPs and Ag_2_S-NPs, and aims to provide a regulatory framework for setting future soil microbial quality guidelines.

## Materials and Methods

**Fig 1:**
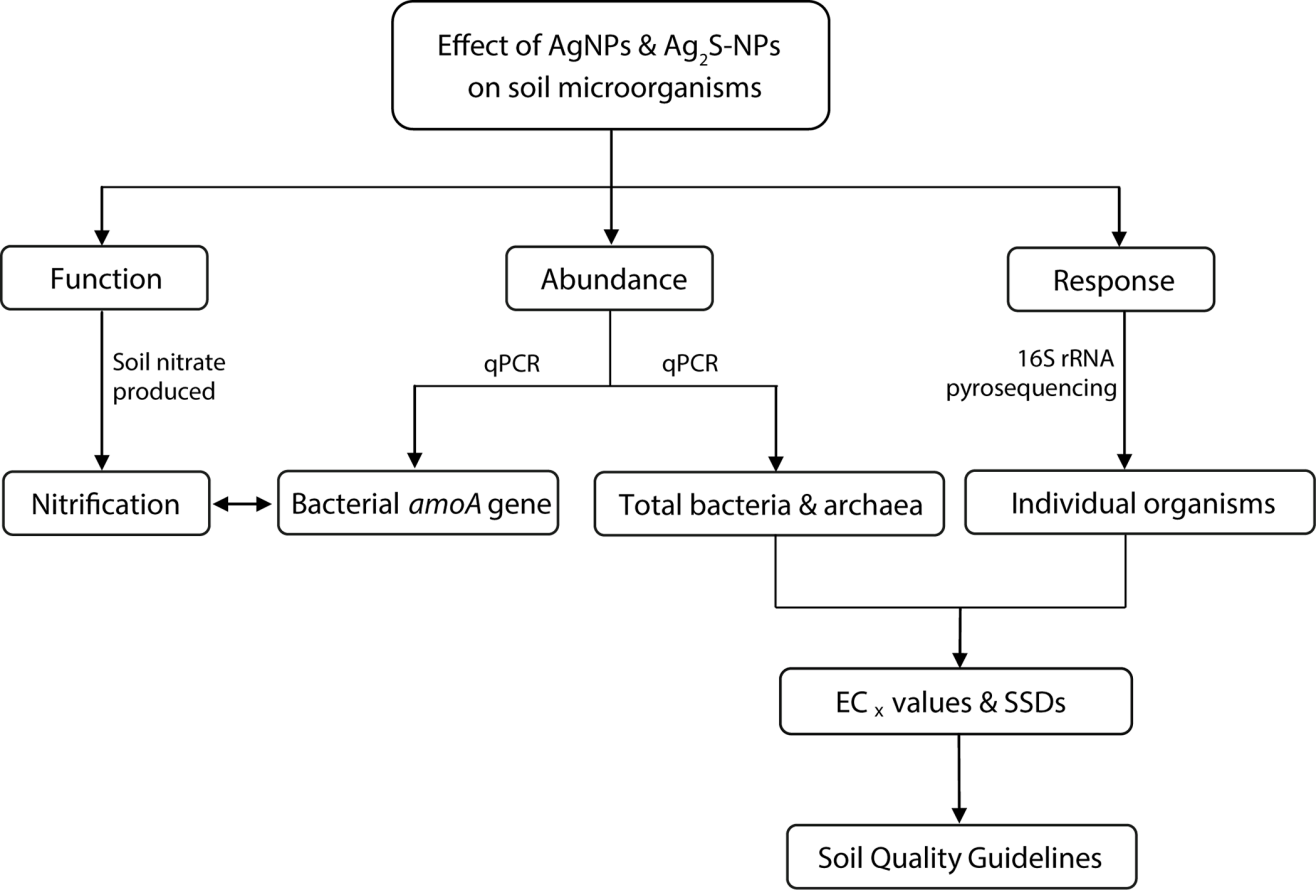
Schematic overview of the study. The techniques that were used to investigate each parameter are shown on the arrows.

An overview of the study is depicted in Fig 1.

### Soil properties

Soil collected from private land in Charleston (South Australia, Australia) (0–20 cm) was used for both the nitrification toxicity test and the microbial DNA sequencing experiment. Permission was given by the owner of the land to collect soil from this location (34°53'42.6"S 138°55'08.3"E). This soil has been characterised previously [22]. The soil was a Chernozem with a slightly acidic pH (pH_CaCl2_ = 5.1) and high organic carbon content (6.9%) (Table A in S1 File). This soil was chosen due to its high concentration of organic carbon; an essential energy source for soil microorganisms. Field moist soil was initially oven dried at 40°C (7 days), sieved (<2 mm) and homogenised prior to experimentation.

### Rates of silver addition

For both experiments, three Ag treatments were applied; AgNO_3_ (Ag^+^), AgNPs and Ag_2_S-NPs. The rates of Ag addition were chosen based on results from a range-finder soil nitrogen transformation test carried out according to OECD Method No. 216 for substrate-induced nitrification [23]. In the nitrification experiment, eight soil spiking rates were used with concentrations ranging from 0.1 to 72 mg Ag kg^-1^, 0.1 to 456 mg Ag kg^-1^ and 0.1 to 2285 mg Ag kg^-1^ for Ag^+^, AgNP and Ag_2_S-NP treatments, respectively. In the sequencing experiment, 14 rates were used with spiking concentrations ranging from 0.1 to 93 mg Ag kg^-1^, 0.1 to 404 mg Ag kg^-1^ and 0.1 to 5590 mg Ag kg^-1^ for the Ag^+^, AgNP and Ag_2_S-NP treatments, respectively. An untreated control (soil with no added Ag), was included in both experiments. The Ag concentration of the control soil was 0.1 mg Ag kg^-1^. Methods of Ag addition and nanoparticles characterisation are presented in S1 Methods.

### Chemical analysis of silver concentrations in soil

Total Ag concentrations of soils were determined using a closed vessel microwave-assisted digestion procedure and analysed by inductively coupled plasma-optical emission spectroscopy (ICP-OES, Optima 7000 DV) and ICP-mass spectrometry (ICP-MS, Agilent 7500ce) as described previously [24] (S1 Methods).

### Soil nitrification toxicity test

#### Experimental set-up

The effect of Ag treatments on soil nitrogen transformation processes were investigated using OECD Method No. 216 for substrate-induced nitrification [23]. Ag^+^, AgNP and Ag_2_S-NP spiked soils were adjusted to 50% of their maximum water holding capacity (MWHC) using ultrapure Milli-Q water and pre-incubated for 7 d. During pre-incubation, soils were maintained at 50% MWHC and stored in the dark at a constant temperature (22°C) with daily aeration. After 7 d, each soil was divided into three replicates (10 g rep^-1^) and amended with powdered lucerne (C:N ratio 13.6:1) at a rate of 5 mg g soil^-1^ (dry weight). Soils were maintained at 50% MWHC for 28 d and stored in the dark with daily aeration.

One subsample was removed from each of the three replicates immediately after lucerne addition (t = 0) and 28 d later (t = 28 d) and extracted with KCl (see S1 Methods). The total production of NO_3_^-^ after 28 d was calculated for each sample by subtracting the NO_3_^-^ soil concentration at t = 0 from that at t = 28 d; this corrects for the NO_3_^-^ present in the control soil and for the NO_3_^-^ that was added with the soluble salt in the AgNO_3_ treatment.

#### Determination of ECx values for nitrification toxicity

For each Ag treatment, dose-response curves were constructed in order to calculate EC_10_, EC_20_ and EC_50_ concentrations, representing decreases of 10%, 20% and 50% in nitrate production. All data were fitted to a four parameter sigmoidal function [22]; the most commonly used dose-response model [25] (Eq 1):

$$y=c+\frac{d-c}{1+{\left(\frac{x}{e}\right)}^{b}}$$ (1)

where *y* = NO_3_^-^ produced as a percentage of the control at Ag concentration *x*; *d* = response in the control (upper asymptote); *c* = minimum effect (lower asymptote), *e =* point of inflection, or the dose when *d–c* is reduced by 50% (EC_50_); and, *b* = slope of the curve around *e* [26].

EC_10_, EC_20_ and EC_50_ values were then interpolated from the fitted curve with a 95% confidence interval.

### Sequencing experiment–impact of silver treatments on the whole soil microbial community

Spiked soils were adjusted to 50% of their MWHC using ultrapure Milli-Q water and stored in the dark at a constant temperature (22°C) for 28 d. The samples were aerated daily and ultrapure Milli-Q water added regularly to maintain the soils at 50% of their MWHC. After 28 d, soils were removed and stored at -20°C for 7 d until DNA extraction.

#### DNA extraction and 16S rRNA amplicon sequencing

DNA was extracted from each soil (0.24 ± 0.02 g, dry soil equivalent) in duplicate using the PowerSoil® DNA Isolation Kit (MoBio Laboratories, Inc., Carlsbad, CA, USA) following the manufacturer’s instructions with minor modification where bead beating was performed using a FastPrep machine (2 x 30 sec at 5 m sec^-1^). Fifty μL of extracted DNA from each replicate was combined to give one representative DNA sample for each treatment. DNA concentrations were determined spectrophotometrically with a NanoDrop ND-1000 (ThermoScientific, USA). DNA (200 ng) from each sample was submitted to the Australian Centre for Ecogenomics (ACE) for 16S amplicon sequencing by Illumina Miseq platform using the 926F (5’-AAACTYAAAKGAATTGACGG-3’) and 1392wR (5’-ACGGGCGGTGWGTRC-3’) primer sets [27].

#### Quantitative polymerase chain reaction (qPCR) analysis

Two quantitative PCR (qPCR) reactions were performed; one to determine the total bacterial and archaeal biomass load (16S rRNA) and a second to analyse the total copy number of the bacterial *amoA* gene. Total bacterial and archaeal biomass load was estimated by qPCR according to Vanwonterghem et al. [28] by ACE with primer sets 1406F (5’-GYACWCACCGCCCGT-3’) and 1525R (5’-AAGGAGGTGWTCCARCC-3’).

DNA from the ammonia oxidizing bacteria (AOB) community and the group specific primers *amoA* 1F (GGGGHTTYTACTGGTGGT) and *amoA* 2R (CCCCTCKGSAAAGCCTTCTTC) were quantified using qPCR [29] based on methods described previously [30] (S1 Methods). The abundance of *amoA* gene copies per g of soil or per ng of DNA, was calculated using data from the standard curve and the DNA yield from the soil extraction. Abundance of the *amoA* gene has been used previously as a basis for determining the toxicity of metal contaminants to nitrification processes in soils [31].

#### Data analysis

Raw paired reads from Illumina sequencing were processed to remove platform specific adapters, primer sequences, and short (< 190bp) and low quality reads (< Phred-33 of 20) using Trimmomatic [32]. All remaining sequences were assembled by Pandaseq [33]. The adapter sequences were removed by FASTQ Clipper of FASTX-Toolkit [34]. Joined high quality sequences were analysed by QIIME v1.8.0 [35] using open-reference operational taxonomic unit (OTU) picking strategy by UCLUST [36] at 1% phylogenetic distance and assigned taxonomy against the Greengenes database (13_05 release), [37, 38]. Singleton and doubleton OTUs were filtered from the OTU table using the command filter_otus_from_otu_table.py in QIIME. Filtered OTUs were imported into R [39] and rarefied to 9,000 sequences per sample using function “rarefu_even_depth” of package phyloseq [40]. Both the 16S rRNA-qPCR results and filtered OTUs table were then imported into Galaxy [41] for gene copy number correction to generate the final absolute abundance of each OTU in each sample using CopyRighter [42]. Rarefaction curves were generated in QIIME to the maximum number of sequences per sample (29,210) against Shannon index (Fig B in S1 File). De-multiplexed sequencing data set were deposited in GenBank under accession number BioProject PRJNA286965.

#### Dose-response curve fitting of OTUs and determination of ECx values

Automated curve fitting of each OTU was performed using the statistical software R (version 3.1.3), with the goal of calculating EC_20_ values from the fitted model. Data were fitted to the log-logistic form of Eq 1 using the R extension package *drc* (version 2.3–96 [43]) i.e. the same dose-response function as used to model the nitrification data (Eq 1). All parameters had the same definition except for *y*, which was equivalent to the total count of single copy 16S gene per μL of sample. Fits were deemed acceptable only when the following criteria were met: 1) *b* > 0, this denotes a negative slope and hence inhibition; 2) *e* (EC_50_) < the maximum spiking concentration; and 3) R^2^ > 0.65. Otherwise, that OTU was excluded from further EC_20_ calculations.

All data were also fitted to a hormesis model (Eq 2) to take into account stimulatory responses at low Ag concentrations [44]. The hormetic model was chosen as ionic Ag has previously been shown to have a stimulatory effect on soil nitrification processes at low concentrations [22].

$$y=c+\frac{d-c+fx}{1+{\left(\frac{x}{e}\right)}^{b}}$$ (2)

where parameters *d*, *c*, *x* and *y* retain the same definition as in Eq 1 and parameters *e* and *b* have no clear biological meaning [44] as they lose their definition as the point of inflection and slope, respectively. The hormesis model includes an additional parameter, *f*, which relates to the initial rate of increase at low doses. Hormesis was deemed statistically significant at the 0.05 probability level if the 95% confidence intervals for *f* (see Eq 3 in S1 Methods) did not intercept zero [45]

Based on the results from curve fitting, OTUs that could not be described by either model (using criteria listed above) were excluded from SSD calculations. For OTUs that were successfully fitted, EC_20_ values were calculated using R (ED.drc function). Note, in this case, EC_20_ refers to the Ag concentration that reduces the absolute abundance of an OTU by 20%. If an OTU could be described by both models, the hormesis model was selected to ensure conservative EC_20_ estimates.

#### Species sensitivity distribution

Calculated EC_20_ values were then used to construct a SSD [46]); here, it is more correctly termed an ‘OTU sensitivity distribution’ (OSD) as OTUs were not assigned to the species level. This distribution plots the cumulative percentage of OTUs affected against the soil concentration of Ag. The distribution of OTU sensitivity was applied to a Burr Type III function [47]. EC_20_ values were fitted to this function using the software package Burrlioz [48] (https://research.csiro.au/software/burrlioz/).

The OSDs were then used to calculate the Ag concentrations that are protective of a specific percentage of OTUs i.e. hazardous concentrations (HC_x_). HC5, HC10 and HC20 values were calculated for each Ag type i.e. Ag concentrations that would affect 5%, 10% and 20% of OTUs, respectively. For each HC value, 95% confidence intervals were calculated by the Burrlioz software using a bootstrap technique [48]. To classify less sensitive OTUs, HC80 values were also calculated.

## Results

### Nitrification experiment

#### Silver decreased soil nitrate production

**Fig 2:**
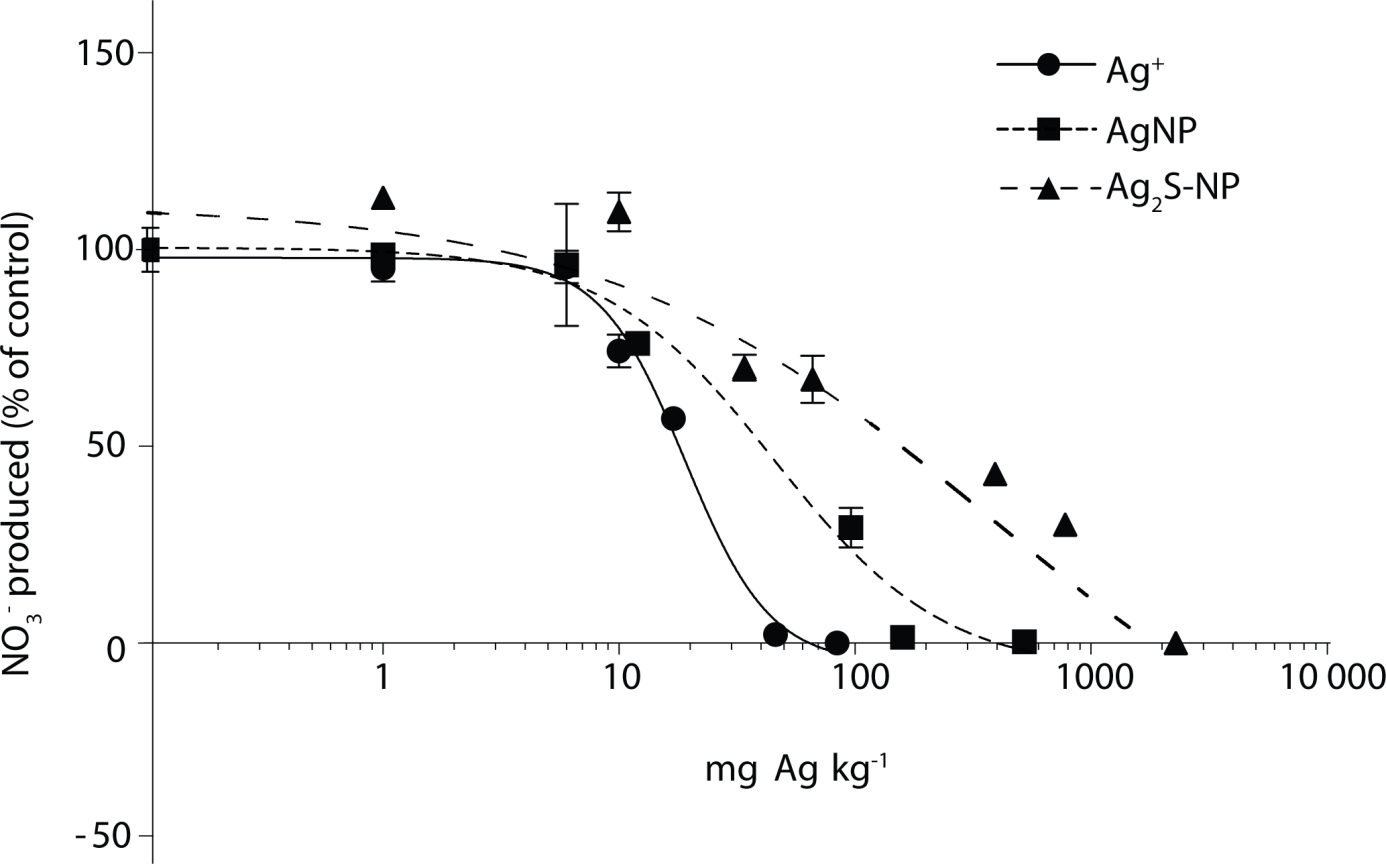
Dose-response curves for NO_3_^-^ production in soil over 28 d. Ionic Ag (Ag^+^–dashed line); Ag nanoparticles (AgNP–short dashed line); and Ag sulfide nanoparticles (Ag_2_S-NP–long dashed line). Mean values (*n* = 3) ± 1 standard deviation are shown. Silver concentrations are on a log_10_ scale.

After 28 d, total nitrate concentrations in the control soil increased from 8.2 mg kg^-1^ to 153.7 mg kg^-1^. Dose-response relationships were observed across all Ag treatments (Fig 2). The EC_10_ concentrations were not significantly different between Ag treatments (*p*>0.05). However, the EC_20_ concentration for Ag_2_S-NP treated soil was significantly greater (*p*<0.05) than that of AgNP and Ag^+^ treatments (Table 1). All EC_50_ concentrations were significantly different (*p*<0.05) and increased in the order Ag_2_S-NPs<AgNPs<Ag^+^ (Table 1). Therefore, it can be concluded that in a Chernozem soil, Ag_2_S-NPs–the most realistic form of Ag in the environment–are significantly less toxic (*p*<0.05) to soil nitrification processes than AgNPs or Ag^+^.

**Table 1:** Silver concentrations (mg Ag kg soil^-1^) that correspond to a 10%, 20% and 50% reduction in soil nitrate production compared to the control (EC_10,_ EC_20_ and EC_50_, respectively). Mean values are shown with 95% confidence intervals in parentheses. For a given EC_x_, significant differences (*p*<0.05) between Ag treatments are indicated by different superscript letters.

EC (mg Ag kg^-1^)	Ag^+^	AgNP	Ag_2_S-NP
EC_10_	8 (6–9)^a^	7 (4–12)^a^	9 (3–21)^a^
EC_20_	11 (9–12)^a^	13 (8–20)^a^	44 (24–72)^b^
EC_50_	19 (17–21)^a^	42 (30–57)^b^	619 (411–899)^c^
*R*^2^	0.98	0.97	0.94

#### Abundance of the bacterial amoA gene

**Fig 3:**
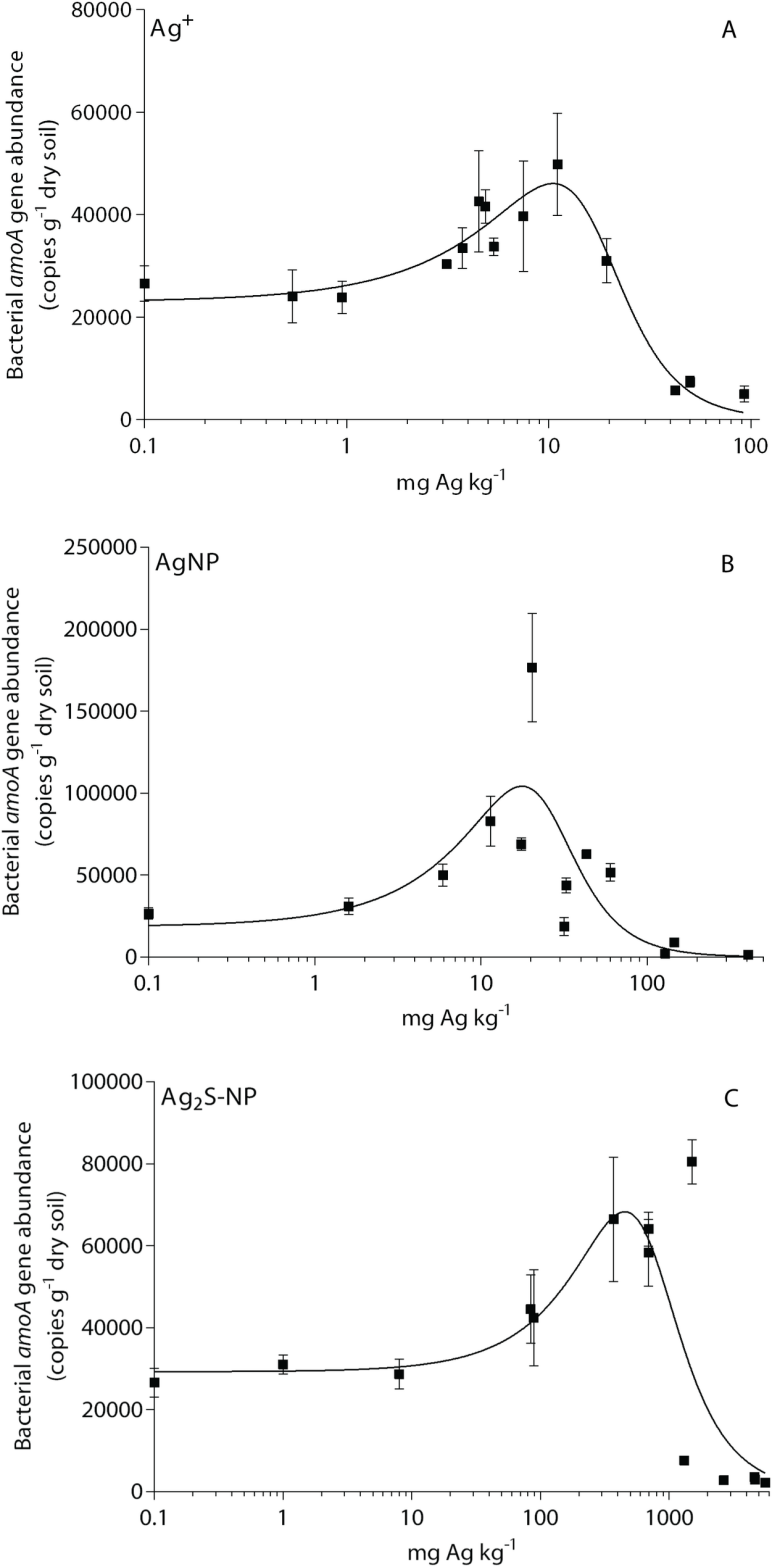
Dose-response curves showing the decrease in total abundance of bacterial *amo*A gene over 28 d. Results are shown for each Ag treatment: (A) ionic Ag (Ag^+^), (B) Ag nanoaparticles (AgNP) and (C) Ag sulfide nanoparticles (Ag_2_S-NP). Mean values (*n* = 4) ± 1 standard deviation are shown. Silver concentrations are on a log scale. All Ag treatments were fitted to the five parameter Brain-Cousens hormesis model as hormesis was significant (*p*<0.05).

The abundance of the bacterial *amoA* gene ranged from 1476 copies g^-1^ to 1.77 × 10^5^ copies g^-1^ (dry soil basis). For all Ag treatments, the abundance of the bacterial *amoA* gene increased at low Ag concentrations and hormesis was significant (Fig 3). The calculated EC_10,_ EC_20_ and EC_50_ values were significantly different between Ag treatments, increasing in the order Ag^+^<AgNP<Ag_2_S-NP (Table B in S1 File). Therefore, the bacterial *amoA* gene was most sensitive to Ag^+^.

### Sequencing experiment

#### Microbial community distribution

The 16S rRNA amplicon sequencing generated 1,608.144 high quality sequences and 461,189 sequences after filtering. Bacterial and archaeal community composition of the control sample was determined from the 16S rRNA sequencing data (Fig A in S1 File). Following the removal of singletons and doubletons, a total of 51,025 OTUs were identified and assigned to 27 different phyla; 26 of which were bacterial and one archaeal. Approximately 7% of OTUs had an unknown classification at any taxonomic level. A very small proportion of OTUs (0.1%) were classified as bacteria but unassigned at the phylum level and thus excluded from Fig A in S1 File. The most dominant phyla in the microbial community–and those accounting for >0.1% of sequences–were Proteobacteria (29%), Actinobacteria (28%) and Firmicutes (21%). Crenarchaeota, the only archaeal phylum, had an abundance of 0.2%. Gemmatimonadetes (5%), Bacteroidetes (3%) and Planctomycetes (3%) were also present in the sample (Fig A in S1 File). The rarefaction curves demonstrate that sequencing depth provided sufficient coverage of the whole community in all samples (Fig B in S1 File).

#### qPCR results for total bacterial and archaeal abundance

**Fig 4:**
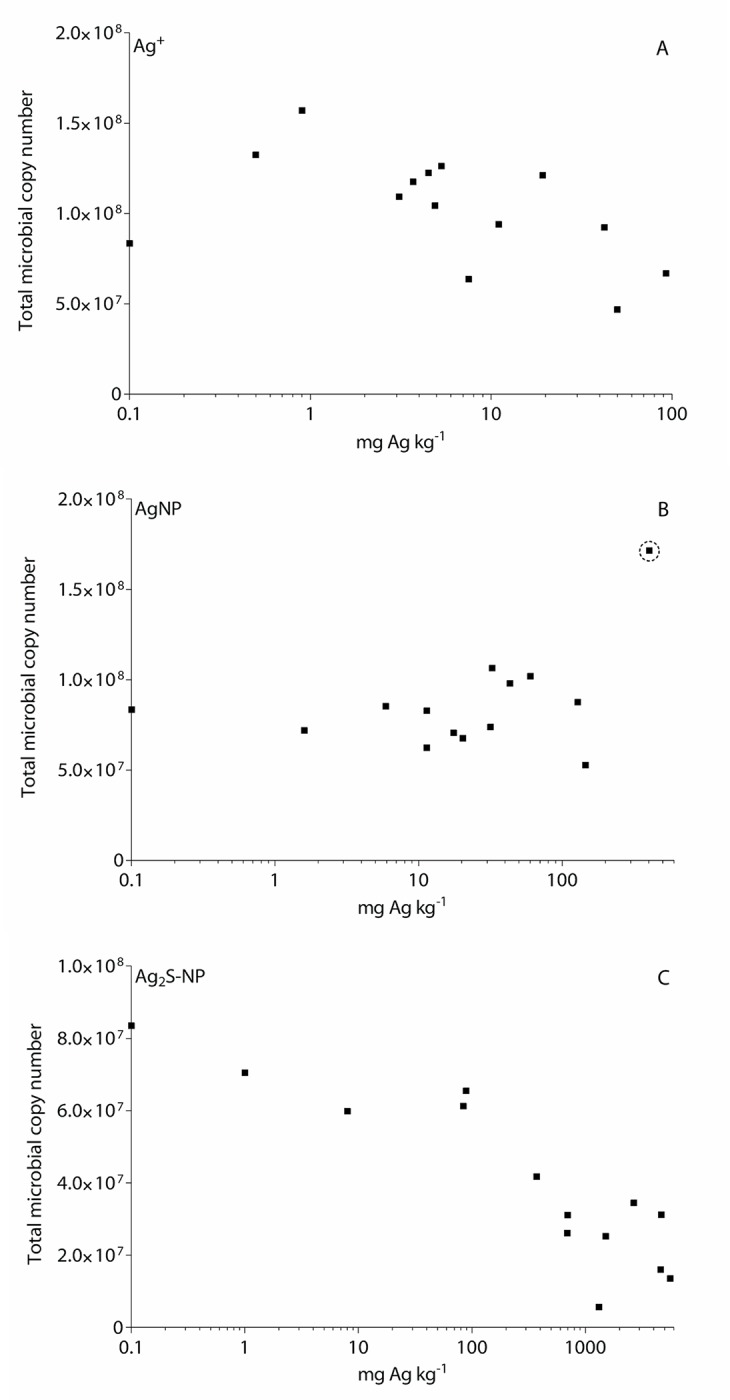
The abundance of bacteria and archaea, as indicated by the number of 16S ribosomal DNA (rDNA) copies measured using quantitative PCR (qPCR). Results are shown for each Ag treatment: (A) ionic Ag (Ag^+^), (B) Ag nanoaparticles (AgNP) and (C) Ag sulfide nanoparticles (Ag_2_S-NP). A likely outlier is circled in the AgNP treatment.

The average 16S gene abundance values for Ag^+^, AgNP and Ag_2_S-NP treatments were 1.03 x 10^8^, 8.69 x 10^7^ and 4.04 x 10^7,^ respectively. There was an observed decrease in bacterial and archaeal abundance with increasing Ag concentrations for Ag^+^ (0–93 mg kg^-1^) and Ag_2_S-NP (0–5590 mg kg^-1^) treatments (Fig 4). While in AgNP treated soil, abundance remained fairly constant with increasing Ag concentrations (0–404 mg kg^-1^).

#### Curve-fitting of dose-response models and non-linear regression analysis of OTUs

Out of the 51,025 OTUs, 47,426 were classified as bacteria, 102 as archaea and 3,497 could not be classified. An OTU needed to appear in at least six samples per Ag type to satisfy degrees of freedom requirements from contestable parameters in Eqs 1 and 2. Operational taxonomic units that did not satisfy these requirements were not assessed further (Table C in S1 File). As a consequence, each Ag treatment had a different number of OTUs that had sufficient observations, and hence could be analysed using non-linear regression (Ag^+^ = 5,444, AgNP = 4,272, Ag_2_S-NPs = 4,259 OTUs). Only a very small number of OTUs (<25) had a stimulation response that could be modelled by the dose-response function.

**Fig 5:**
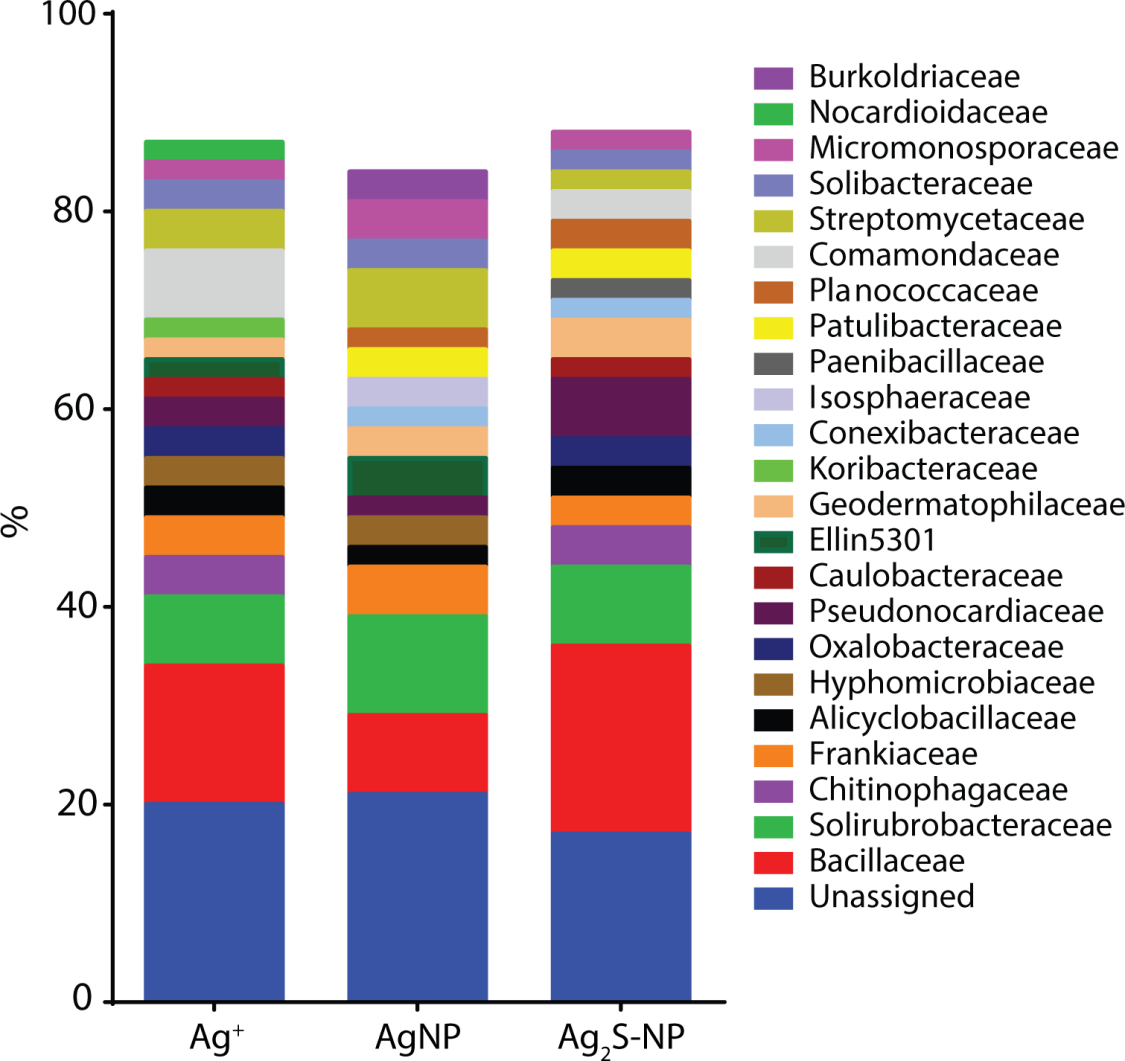
Taxonomy of the OTUs that were successfully fitted to the dose-response models. For clarity, only the bacterial families that contributed ≥2% to the overall distribution are shown. Unassigned OTUs are unclassified at the family level but are classified at higher taxonomic levels.

Multiple dose-response curves were successfully constructed for each Ag treatment (examples shown in Figs D and E in S1 File); however, the number of curves differed between each Ag type (Ag_2_S-NP = 498, Ag^+^ = 390, AgNP = 146 OTUs) (Table D in S1 File). When comparing the taxonomy of the fitted OTUs between Ag treatments, similar distributions of phyla and families were observed (Table E in S1 File and Fig 5, respectively). In all Ag treatments, the microbial communities were dominated by key phyla including Actinobacteria, Proteobacteria and Firmicutes (78–85%). Other phyla accounted for less than 10% of the microbial community. *Nitrospirae* and *Elusimicrobia* were only fitted in the Ag^+^ treatment (0.3%). For each Ag treatment, the distribution of the most abundant families is given in S1 Table.

When OTUs were assigned to the family level, minor differences between Ag treatments were observed (Fig 5). For example, in the AgNP treatment, no OTUs were affiliated with *Chitinophagacea*e, while in the Ag_2_S-NP and Ag^+^ treatments, this family accounted for approximately 5%. The abundance of *Bacillaceae* was also less in the AgNP treatment (8%) compared to Ag^+^ and Ag_2_S-NP treatments (14% and 19%, respectively). In the Ag^+^ treatment, *Comamonadaceae* was the third most dominant family comprising 7% of sequences. However, it was less dominant in the Ag_2_S-NP treatment (3%) and was absent in AgNP treatment.

#### Calculation of toxicity values (EC20) and hazardous concentrations

**Fig 6:**
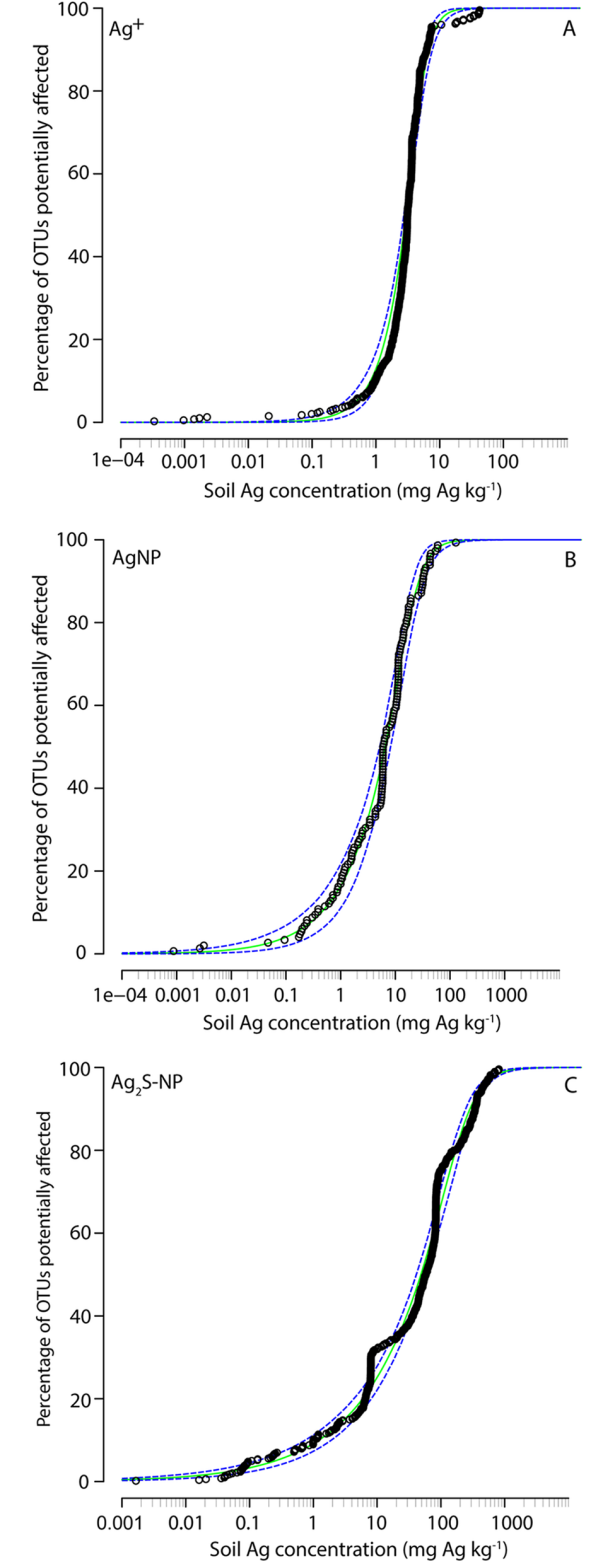
Operational taxonomic unit (OTU) sensitivity distributions (OSD) comparing the sensitivity of OTUs to each Ag treatment. Results are shown for each Ag treatment: (A) ionic Ag (Ag^+^), (B) Ag nanoaparticles (AgNP) and (C) Ag sulfide nanoparticles (Ag_2_S-NP). Each data point corresponds to the Ag concentration that decreased the absolute abundance of a specific OTU by 20% (EC_20_). Data were fitted to a Burr Type III function, where the fitted function is shown in green and 95% confidence intervals are indicated by the blue dashed line.

For OTUs that are termed ‘fitted OTUs’ in the preceding sections, their response to Ag can be described by either the sigmoidal dose-response function (Eq 1) or the hormesis function (Eq 2)–or both (Table D in S1 File). For each Ag treatment, toxicity values (EC_20_) were determined for these OTUs and plotted on separate OSDs for each Ag type (Fig 6). The distribution of EC_20_ values followed a sigmoidal shape.

For HC5 and HC10 values, there were no significant differences between Ag treatments (*p*>0.05) (Table 2). However, at the least protective HC value (HC20, 80% protection), Ag_2_S-NPs were significantly less toxic than Ag^+^ and AgNPs (*p*<0.05).

**Table 2:** Hazardous concentrations (HC) for ionic Ag (Ag^+^), Ag nanoparticles (AgNP) and Ag sulfide nanoparticles (Ag_2_S-NP) at which 95%, 90% and 80% of soil OTUs are protected (HC5, HC10 and HC20, respectively). Upper and lower 95% confidence intervals are shown in parentheses. Hazardous concentrations at which only 20% of soil OTUs are protected (HC80) were calculated to define the less sensitive OTUs. For a given HC, significant differences (*p*<0.05) between Ag treatments are indicated by different superscript letters.

Hazardous concentration (mg Ag/kg)	Silver type		
	Ag^+^	AgNP	Ag_2_S-NP
HC5	0.49^a^	0.14^a^	0.25^a^
	(0.32–0.73)	(0.056–0.35)	(0.13–0.47)
HC10	0.83^a^	0.44^a^	1.2^a^
	(0.61–1.1)	(0.22–0.86)	(0.76–2.0)
HC20	1.4^a^	1.4^a^	5.9^b^
	(1.2–1.7)	(0.89–2.2)	(4.4–8.1)
HC80	5.1^a^	17.0^b^	171.0^c^
	(4.7–5.6)	(14–22)	(144–203)
Number of data points in SSD curve	390	146	498

#### Taxonomy of silver-sensitive and silver-tolerant OTUs

Across all Ag treatments, the most sensitive OTUs (EC20<HC5) were predominantly from the *Bacillaceae* family. Other sensitive OTUs were affiliated to family *Frankiaceae* and *Comamonadaceae* for Ag^+^ (6 OTUs of 20); *Planococcaceae*, *Thermomonosporaceae* and *Micromonosporaceae* for AgNP (3 OTUs of 5); and *Pseudonocardiaceae* and *Micromonosporaceae* for Ag_2_S-NPs (8 OTUs of 34). In general, there was no significant linear correlation in EC_20_ values for Ag, AgNP, and Ag_2_S-NPs in OTUs for which EC_20_ values could be identified (*p>*0.05).

Of the total number of OTUs fitted to dose-response models, zero, two and four OTUs were unassigned at all taxonomic levels for the Ag^+^, AgNP and Ag_2_S-NP treatments, respectively. Therefore, in the proceeding discussion, ‘unassigned’ refers to OTUs that are classified at higher levels but not at lower taxonomic levels (i.e. not OTUs without any taxonomic information). Less sensitive OTUs, those in the upper part of the OSD (EC_20_>HC80), were assigned to consistent families. Again, *Bacillaceae* was the most dominant family for Ag_2_S-NP OTUs (26%), followed by *Chitinophagaceae* (13%), *Solirubrobacteraceae* (16%) and OTUs that were not classified at the family level (8%). In the Ag^+^ treatment, unassigned OTUs (those not classified at family level) were the most abundant (22%), followed by *Solirubrobacteraceae* (10%), *Frankiaceae* (9%) and *Ellin5301* (7%). Similarly, for AgNPs, dominant OTUs were either unassigned or classified as *Bacillaceae*, *Geodermatophilaceae* or *Streptomycetaceae* (22%, 15%, 11% and 7%, respectively).

## Discussion

### Silver treatments decrease soil nitrate production

This study is the first, to our knowledge, to investigate the effects of Ag_2_S-NPs on nitrification in a natural soil. Sulfidised AgNPs were less toxic to soil nitrification processes than Ag^+^. Following sulfidation, both the toxicity and bioavailability of AgNPs are reduced [7, 49, 50], most likely due to low solubility of Ag_2_S (K_sp_ = 1.6 x 10^−49^ [51]). In addition to sulfidation, NP size and/or other physico-chemical properties may have influenced the toxicity of AgNPs and Ag_2_S-NPs. However, sulfidation is likely to have had the greatest effect given that it substantially reduces the release of free Ag^+^.

EC_50_ concentrations were significantly lower for Ag^+^ compared to AgNPs, demonstrating that AgNPs were less toxic to the soil nitrification process than Ag^+^. However, EC_10_ and EC_20_ concentrations were not significantly different (*p*>0.05) between AgNP and Ag^+^ treatments. This may due to greater uncertainty in the tails of the distribution. In addition, different mechanisms may have been operating at different Ag concentrations. At low Ag concentrations, AgNP dissolution may have been rapid and therefore any effects would be similar to that of soluble Ag (Ag^+^). Conversely, at higher Ag concentrations (i.e. EC_50_), the rate of AgNP dissolution may have been slower, meaning that other mechanisms would have greater influence on AgNP behaviour in soil. Heterocoagulation of AgNPs with natural soil colloids (e.g. clay particles) [52] is one such mechanism that can decrease dissolution and thus limit the release of toxic Ag^+^ [53]. Similarly, VandeVoort and Arai [54]), demonstrated a correlation between the adsorption of AgNPs to soil surfaces and the extent of AgNP toxicity to soil denitrification processes. Uncoated AgNPs had the least affinity for soil surfaces and were toxic to soil denitrifcation processes at 100 mg Ag L^-1^, whereas PVP-coated AgNPs had greater affinity for soil surfaces and were not toxic at this concentration [54]).

In the current study, the Ag^+^ EC_50_ for nitrification was less than that calculated previously for the same soil (19 vs 47 mg Ag kg^-1^ soil [22]). It is unclear why this discrepancy occurred. One explanation may lie in the different models that were used to fit the data. Previously, a hormetic model was used whereas in the current experiment, a regular sigmoidal model was used as hormesis was not significant. Differences in sample preparation and changes in the microbial community composition over time (during soil storage) may have also contributed to the observed differences.

Few studies have investigated the effect of AgNPs on soil nitrification processes. Instead, most nitrification studies have focused on either wastewater or specific nitrifying bacteria in culture media. For example, the abundance of nitrifying bacteria in sludge significantly decreased when exposed to AgNPs at 40 mg Ag L^-1^ [55], while at lower Ag concentrations (2.5 mg L^-1^), nitrate production in sludge was not affected [56]. In culture media, nitrate production decreased by 90% when the model AOB, *Nitrosomonas europaea*, was exposed to AgNPs (20 mg Ag L^-1^) [57]. Only one previous study has used a natural soil to investigate the impacts of AgNPs on nitrification [58]. Silver NPs were found to be more toxic to nitrification than Ag^+^ when added to a soil slurry at 1 mg Ag L^-1^ [58]. Conversely, in culture media, Ag^+^ was 48-times more toxic to nitrogen-cycling bacteria than AgNPs [59].

Overall, findings from the current study suggest that the risk of Ag-based NPs (especially Ag_2_S-NPs) to soil nitrification is overestimated (and conservatively covered) by the risk of ionic Ag^+^ in soil environments. The results also demonstrate the concentration ranges over which this soil microbial process will be affected by Ag (Fig 2).

#### The toxicity of silver nanoparticles to soil microbial processes is controlled by multiple factors

Nitrification capability was initially a key focus of the study, and dose-response curves for the effect of Ag on soil nitrification and bacterial *amoA* gene abundance were developed (Table 1 and Table B in S1 File). For example, the abundance of the bacterial *amoA* gene increased at low Ag concentrations (hormesis), whereas the production of soil nitrate was not stimulated at low concentrations for any Ag treatment. Silver has been shown to have a stimulatory effect on nitrifying genes (*amoA* and *amoC2*) in *N*. *europaea* at low concentrations (2.5 μg Ag L^-1^) [59]. The mechanisms behind this hormetic effect are unknown but may be a related to a stress response which increases the rate of respiration [59]. Silver has also been shown to have a hormetic effect on plant biomass (*Carex lurida*) [60] and soil nitrification in various soil types [22].

In the current study, although abundance of the *amoA* gene increased, the composition of the whole community also changed. This highlights the need to use a variety of approaches when investigating the effect of contaminants on soil microbial communities. Some of the analyses should include: qPCR, for abundances of total or specific functional groups; RT-PCR, for active populations; amplicon sequencing, for determining community composition; measurement of the abundance of specific genes, e.g. *amoA*; and, measurement of the effects on specific functions (e.g. nitrate production).

In the incubation study, the observed toxicity to nitrification could not be directly related to the response of specific OTUs. Five genera associated with nitrification processes were identified in the overall community; *Nitratireductor*, *Nitrobacter*, *Nitrosospira*, *Nitrosovibrio* and *Nitrospira*. However, each of these genera was affiliated to <0.1% of OTUs. Furthermore, of the OTUs successfully fitted to dose-response functions, only seven were assigned to these genera (all *Nitrosovibrio*) (Table H in S1 File). OTUs affiliated to other known nitrification genera were not present (e.g. *Nitrosopumilus*, *Nitrosomonas*). Therefore, to identify the specific members of the nitrifying community that were affected, a more in depth analysis could be performed using a functional gene (*amo*A) sequencing approach. Soil nitrification processes are controlled by a very complex autotrophic community [61], with the phylogenetic affiliation of many ammonia-oxidising archaea (AOA), AOB and nitrite-oxidising bacteria (NOB) still unknown [62].

### Microbial community distribution

Overall, the community composition is consistent with previous observations in Australian soils [63], where Actinobacteria and Proteobacteria are the most dominant phyla. However, the abundance of Firmicutes (21.3%) was relatively high compared to other studies (e.g. 0.9% [64]) and Acidobacteria (2.9%) was slightly lower than expected (e.g. 13.8% [64]). Although Firmicutes are usually considered a low-abundance phylum [65], they have been found to dominate the bacterial soil community in loamy-sand agricultural soils [66]. The abundance of Firmicutes has also been shown to increase in the presence of chitin in Chernozem soils [67].

### Estimated hazardous concentrations of silver treatments to microbial communities

Overall, hazardous concentrations of AgNPs and Ag_2_S-NPs to soil OTUs were less than or equal to that of Ag^+^. When considering a protection level of 80%, Ag_2_S-NPs were significantly less toxic than Ag^+^ or AgNPs. In contrast, a recent mesocosm study suggested that sulfidised AgNPs (applied as AgNPs+sludge) were more toxic than Ag^+^ to soil microbial biomass and function (specifically N_2_O flux) [68]. However, after 50 d, no significant differences were observed between Ag treatments and the control (*p*>0.05). In biosolids-treated soils, the concentration of AgNPs has been reported to be between 0.1–1 μg kg^-1^, with a yearly increase of 110 ng Ag kg^-1^ [69]. All HC_x_ values calculated in the current study (0.14–5.9 mg Ag kg^-1^ soil) exceeded this concentration range. Therefore, based on these findings and predicted Ag soil concentrations, AgNPs and Ag_2_S-NPs appear to pose a low risk to whole soil microbial communities.

Currently, geogenic concentrations of Ag in soils (0.01–1 mg kg^-1^ [70]) are much greater than that for predicted loadings of Ag-based NPs; however, this will be exceeded in ~90 years according to current predictions. Therefore, based on the calculated HC5 and HC10 values, soil microbial communities are potentially at risk in some of these soils. However, it is important not to overestimate this risk based on these soil concentrations alone as the bioavailability of Ag (and other metals) in soils is affected by many factors, including speciation, effects of aging on speciation and, the physical and chemical properties of the soil [71, 72].

### Limitations, implications, and future recommendations for the risk assessment of transformed silver nanoparticles in soils

A limitation of the dose-response curve-fitting procedure is that EC_20_ values were estimated from the upper asymptote of the curve and not from the control value. As a result, EC_20_ values for a small number of fitted OTUs (<10 for each Ag treatment) were less than the control concentration i.e. between 0–0.1 mg Ag kg^-1^. This is a numerical artefact that can be attributed to the increased sensitivity of these OTUs; they are possibly affected immediately by Ag addition and no dose can be considered ‘safe’. Indeed, it is likely that a number of OTUs would follow this response due to random effects. However, further analysis of these OTUs was not performed as it is out of the scope of this study.

Using published acute toxicity values, SSDs have recently been constructed for aquatic freshwater species exposed to common nanomaterials (including AgNPs) [73]. However, similar data are not available for soil organisms exposed to any nanomaterial. In fact, for all toxicants, very few studies have applied the SSD methodology to terrestrial organisms due to the lack of toxicity data for soil species at all trophic levels [74, 75]. Previously constructed SSDs have only considered soil invertebrates (e.g. nematodes and annelids) and plants (monocots and dicots); microorganisms have not been modelled except when included in multi-trophic level SSDs due to the difficulties in culturing soil microorganisms.

Our study is the first to construct an SSD (OSD) for microorganisms exposed to nanomaterials using DNA sequencing without the need for culturing soil microorganisms. An important limitation, however, is that due to stochastic effects, the results are only applicable to the soil type tested (Chernozem). Different OSDs will need to be constructed for other soil types that would have different microbial community distributions and soil properties (soil pH, clay content and concentration of organic matter) that would control Ag toxicity [22, 76]. This is a worthwhile subject for future studies. Collectively, data from different soil types and analysis of systematic changes would enable better mechanistic understanding of the link between function, toxicity, and phylogeny. Such a comparison could also identify members of the soil microbial community that are commonly affected by AgNPs across soil types and environments. SSD development is only the first step in the ecological risk assessment of AgNPs and Ag_2_S-NPs. Further studies are needed to establish if the approach is applicable across a range of soil types and to determine the functional role of sensitive microorganisms.

## Supporting Information

S1 File Supplementary Figures and Tables. (PDF)

S1 Methods Supplementary Methods. (PDF)

S1 Table Overall Taxonomy of OTUs. Dominant families fitted to each dose-response model.
(XLSX)
