# Supplementary material for: Quantifying the Sensitivity of Soil Microbial Communities to Silver Sulfide Nanoparticles Using Metagenome Sequencing
Source: PLoS One. 2016 Aug 30;11(8):e0161979. doi: 10.1371/journal.pone.0161979 (PMC5004803; doi:10.1371/journal.pone.0161979)
Supplement: S1 File — (PDF) [file pone.0161979.s001.pdf]

S1

Figures and Tables

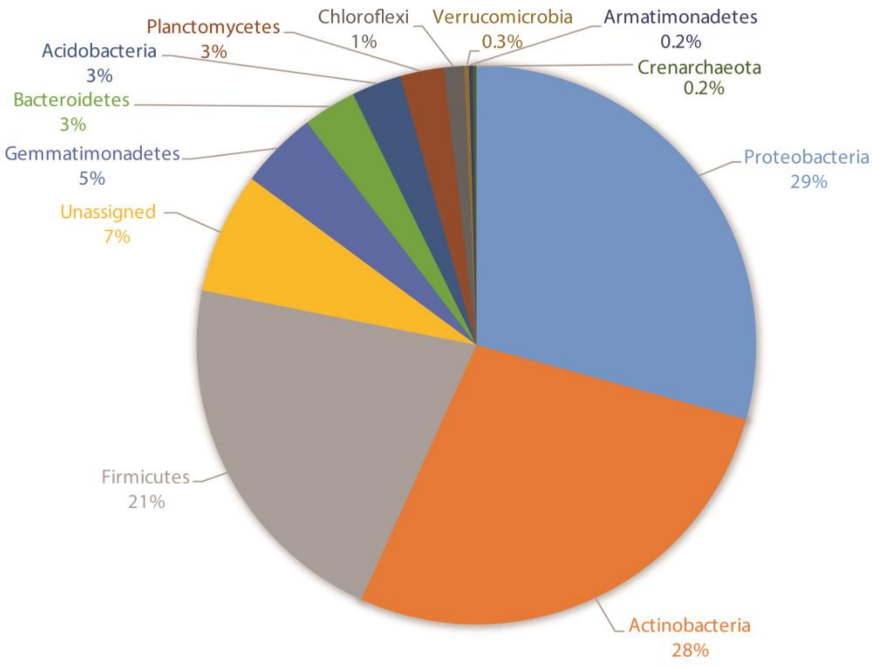

Figure A. Distribution of bacterial and archaeal phyla in Chernozem soil collected from Charleston, South Australia. Phyla accounting for > 0.1% of sequences are shown. Sequences were clustered into OTUs at 99% similarity. Unassigned OTUs have an unknown classification at any taxonomic level.

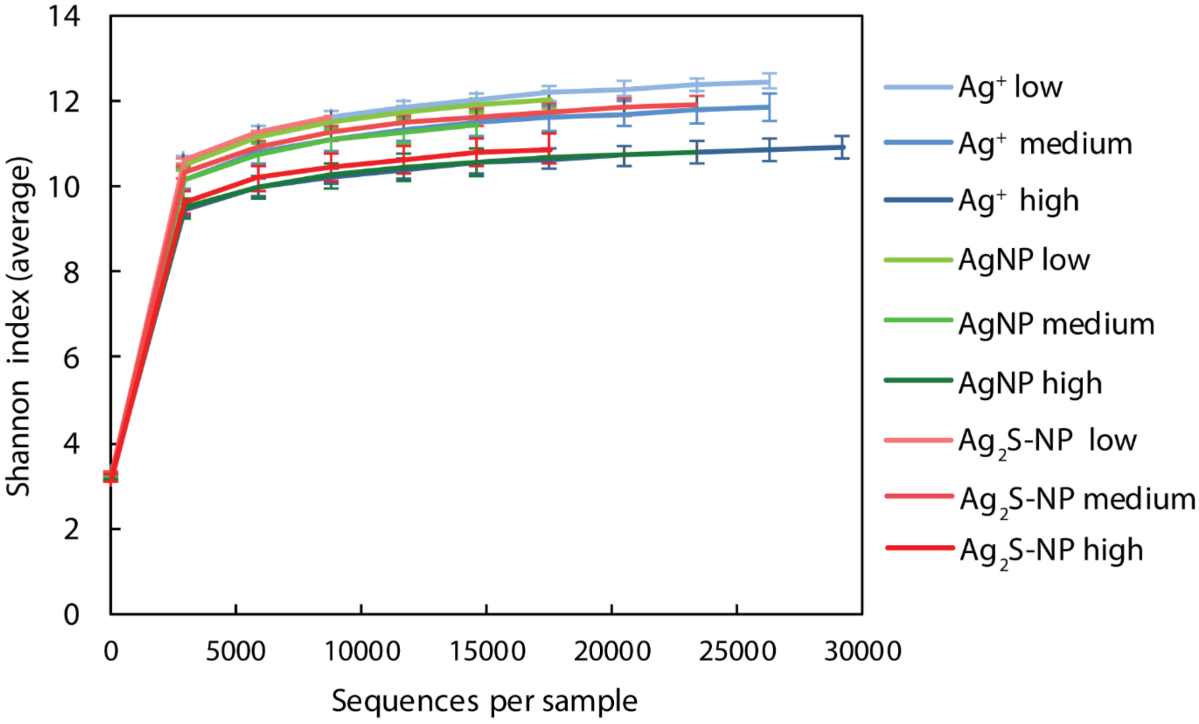

Figure B. Rarefaction curves for ionic Ag (Ag^+); Ag nanoparticles (AgNP); and Ag sulfide nanoparticles (Ag_2S-NP). Silver concentrations (mg Ag kg^{-1}) were defined as follows: Ag^+ low (0–10), medium (11–20), high (21–100); AgNP low (0–15), medium (16–50), high (51–405); Ag_2S-NP low (0–100), medium (101–1000), high (1001–6000).

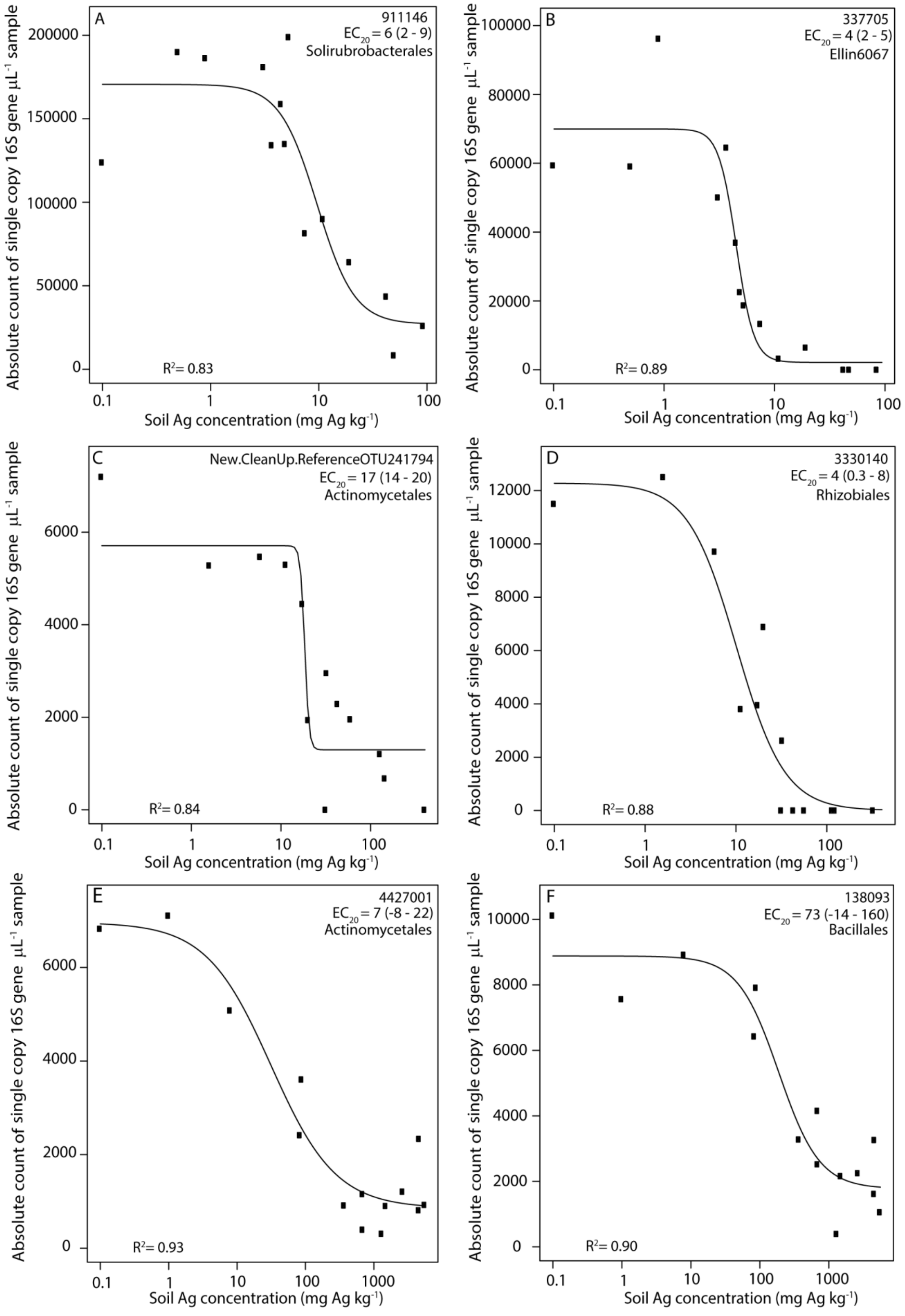

Figure C. Dose-response plots of the absolute abundance of selected OTUs following 28 d soil incubation. Selected plots are shown for ionic Ag (Ag^+ – A,B); Ag nanoparticles (AgNP – C,D); and Ag sulfide nanoparticles (Ag_2S-NP – E,F) . The OTU identity, assigned order and the calculated EC_{20} values (with 95% confidence intervals) are shown at the top right of each plot. See Table F in S1 File (below) for estimates of the fitting parameters and associated errors.

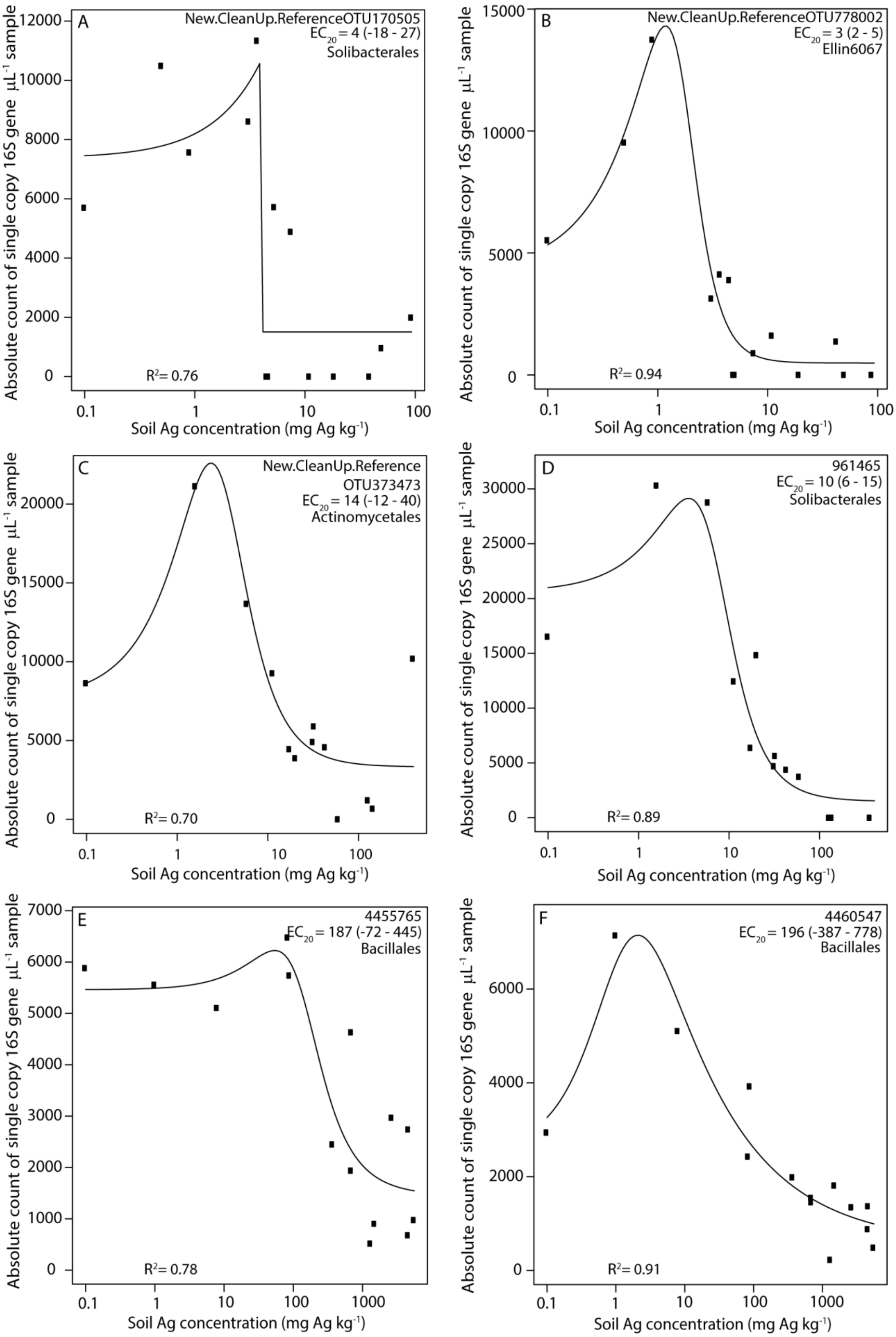

Figure D. Dose-response plots of the absolute abundance of selected OTUs that demonstrated significant hormesis ($p \leq 0.05$) following 28 d soil incubation. Selected plots are shown for ionic Ag (Ag^+ – A,B); Ag nanoparticles (AgNP – C,D); and Ag sulfide nanoparticles (Ag_2S-NP – E, F) . The OTU identity, assigned order and the calculated EC_{20} values (with 95% confidence intervals) are shown at the top right of each plot. See Table G in S1 File (below) for estimates of the fitting parameters and associated errors.

Table A. Physicochemical properties of the soil that was used in both experiments (collected from Charleston, South Australia).

$pH_{(CaCl_2)}$	$pH_{(H_2O)}$	Total Ag (mg kg^{-1})	Organic C (%)	MWHC (%)	CEC (cmol$^+$ kg^{-1})	Particle size analysis Clay %	Silt %	Sand %
5.1	6.6	< 0.04	6.9	51	12.0	14	12	63

CEC = cation exchange capacity; organic C = organic carbon; MWHC = maximum water holding capacity

Table B. Silver concentrations (mg Ag kg soil^{-1}) that correspond to a 10%, 20% and 50% reduction in total copy number of the bacterial *amoA* gene. Mean values are shown ($n = 4$) with 95% confidence intervals in parentheses. Significant differences between Ag treatments ($p < 0.05$) for each ECx value are indicated by different superscript letters.

EC (mg Ag kg^{-1})	Ag^+	AgNP	Ag_2S-NP
EC_{10}	26 (21 – 35)^a	73 (47 – 128)^b	1636 (1235 – 2530)^c
EC_{20}	28 (22 – 38)^a	77 (48 – 138)^b	1788 (1328 – 2624)^c
EC_{50}	35 (26 – 51)^a	98 (52 – 186)^b	2503 (1687 – 4125)^c
r^2	0.64	0.50	0.58

Table C. The number of OTUs that were removed from the dataset prior to non-linear regression analysis. OTUs were removed if they were only counted between 0 and 5 times in each silver treatment. The remaining OTUs were used in non-linear regression analysis.

Count of OTUs	Number of OTUs Ag^+	AgNP	Ag_2S-NP
Total OTUs initially	51025	51025	51025
0	5882	8827	10214
1 (singletons)	14401	16411	15078
2 (doubletons)	12970	11887	11315
3 (tripletons)	7033	5433	5720
4	3218	2663	2742
5	2077	1532	1697
Remaining OTUs*	5444	4272	4259

Table D. The number of OTUs that were fitted to each model type for each Ag treatment. Operational taxonomic units (OTUs) that could be fitted to the four parameter log-logistic sigmoidal model (LL.4) and the Brain-Cousens hormesis model (BC.5), were plotted on the OTU-sensitivity curve. OTUs that were fitted to the models 'LL.4 (inhibition)', 'BC.5' and 'LL.4 + BC.5' were plotted on the OTU sensitivity distribution.

Fitting model	Ag^+	AgNP	Ag_2S-NP
LL.4 (inhibition)	319	68	273
BC.5	44	46	106
LL.4 + BC.5	27	32	119
LL.4 (stimulation)	22	15	15
Linear regression (slope > 0)*	111	400	144
Linear regression (slope < 0)*	241	43	284
Non-significant fits#	4680	3668	3318
Total	5444	4272	4259

*Linear slope is significantly < or > zero ($p < 0.05$).

#Non-significant fits were OTUs that could not be fitted to either dose response function (LL.4 or BC.5), or when fitted to the linear model, the slope was not significantly different from zero.

Table E. Distribution of phyla that were successfully fitted to the dose-response models and subsequently used in the OTU sensitivity distribution. The abundance of each phylum is displayed as a percentage of the total count of all phyla for each Ag treatment.

Phylum	Ag^+	AgNP	Ag_2S-NP
Actinobacteria	31.5%	48.3%	41.4%
Proteobacteria	28.5%	19.0%	15.5%
Firmicutes	18.7%	13.6%	28.9%
Acidobacteria	5.4%	4.8%	4.2%
Bacteroidetes	4.1%	0.7%	4.2%
Planctomycetes	4.1%	4.1%	2.4%
Gemmatimonadetes	4.1%	6.1%	1.4%
Chloroflexi	1.0%	1.4%	0.6%
Armatimonadetes	0.8%	0.7%	0.2%
Unassigned	0.8%	0.7%	0.8%
Verrucomicrobia	0.5%		0.2%
Elusimicrobia	0.3%		
Nitrospirae	0.3%		
Acidobacteria			
Crenarchaeota		0.7%	0.2%

Table F. Results of dose-response curve fitting for OTUs presented in Figure C in S1 File (above). The estimated values of the fitting parameters *b*, *c*, *d* and *e* are shown with their 95% confidence intervals.

OTU	Ag$^+$			AgNP	Ag$_2$S-NP	
	911146	337705	New.CleanUp. Reference OTU241794	3330140	4427001	138093
b	3	5	18	2	1	1
Lower CI	-1	-3	-18	0.4	0.2	0.1
UpperCI	6	14	54	3	2	3
c	27004	2585	1296	0	854	1775
Lower CI	-23016	-9153	394	-2345	134	412
Upper CI	77024	14323	2197	2345	1575	3138
d	170627	70417	5708	12280	6962	8881
Lower CI	136449	54683	4641	9193	5495	7381
Upper CI	204806	86151	6776	15368	8428	10381
e	10	4	18	10	31	188
Lower CI	2	4	16	4	-12	19
Upper CI	17	5	21	16	75	357
EC_{20}	6	3	17	4	7	73
Lower CI	2	2	14	0	-9	-14
Upper CI	9	5	20	8	22	160
R^2	0.83	0.89	0.84	0.88	0.93	0.90

Table G. Results of dose-response curve fitting for OTUs presented in Figure D in S1 File (above). The estimated values of the fitting parameters *b*, *c*, *d*, *e* and *f* are shown with their 95% confidence intervals.

OTU	Ag+		AgNP		Ag$_2$S-NP	
	New.CleanUp. Reference OTU170505	New.CleanUp. Reference OTU778002	New.CleanUp. Reference OTU373473	961465	4455765	4460547
b	5640	4	2	2	2	1
Lower CI	5618	1	0	1	0	1
UpperCI	5663	1	0	1	0	1
c	1506	487	3327	1488	1445	557
Lower CI	1483	-737	-1018	-3974	-569	-1012
UpperCI	1529	1711	7672	6949	3460	2127
d	7371	4157	7582	20594	5461	2544
Lower CI	7349	911	-2348	12037	3722	529
UpperCI	7394	7404	17512	29151	7200	4560
e	4	2	3	7	135	1
Lower CI	-19	1	-1	2	-301	0
UpperCI	27	3	7	11	571	3
f	823	11774	10642	3986	28	7902
LowerCI	801	3887	-5449	-2609	-141	146
UpperCI	846	19661	26732	10580	198	15658
EC_{20}	4	3	14	10	187	196
Lower CI	-19	2	-12	5	-72	-387
Upper CI	27	5	40	15	445	778
R^2	0.76	0.94	0.70	0.89	0.78	0.91

Table H. Genera commonly associated with nitrification processes that were identified in the sequencing data. The count of OTUs that were assigned to these genera are given for the overall microbial community and for each Ag treatment based on the fitted model.

	Nitratireductor	Nitrobacter	Nitrosospira	Nitrosovibrio	Nitrospira
Overall community	1 (<0.1%)	1 (<0.1%)	25 (<0.1%)	45 (<0.1%)	4 (<0.1%)
Ag^+					
OSD				1	
drc_inc					
linear_inc					
linear_dec				2	
AgNP					
OSD					
drc_inc					
linear_inc					
linear_dec				1	
Ag_2S-NP					
OSD					
drc_inc					
linear_inc					
linear_dec				3	

OSD = OTUs that were used in the OTU sensitivity distribution (i.e. successfully fitted to the dose-response model or hormesis model); drc_inc = OTUs fitted to an increasing dose-response model; linear_inc/dec = OTUs that had a linear slope significantly greater than or less than zero ($p < 0.05$).
