# Supplementary material for: Quantifying the Sensitivity of Soil Microbial Communities to Silver Sulfide Nanoparticles Using Metagenome Sequencing
Source: PLoS One. 2016 Aug 30;11(8):e0161979. doi: 10.1371/journal.pone.0161979 (PMC5004803; doi:10.1371/journal.pone.0161979)
Supplement: S1 Methods — (PDF) [file pone.0161979.s002.pdf]

S1

Supplementary Methods

1.1. Silver addition to soil

Silver NPs were added to soil as a suspension. For soils with spiking concentrations between 1 and 200 mg Ag kg^{-1}, a AgNP suspension was used that has been described previously [1]. Briefly, 0.1 g of PVP-coated AgNP powder (Nanoamor) was added to 50 mL of ultrapure Milli-Q water, sonicated (90 W, 3 min) and then centrifuged (2200 *g*, 15 min). For the highest AgNP spiking rate, a more concentrated AgNP suspension was used. This concentrated suspension was prepared by weighing AgNP (0.019 g) into a 10 mL centrifuge tube, adding ultrapure Milli-Q water (7.65 mL) and probe sonicating (90 W) for 20 sec before adding to soil (30 g).

For Ag_2S-NP treatments that were between 1 and 100 mg Ag kg^{-1}, a previously described Ag_2S-NP suspension was used [2]. Silver sulfide NP treatments that were greater than 500 mg Ag kg^{-1} were spiked with a more concentrated Ag_2S-NP suspension that was prepared separately for each treatment. These concentrated suspensions were prepared by weighing increasing amounts of Ag_2S-NP powder into 10 mL centrifuge tubes, adding ultrapure Milli-Q water (7.65 mL) and probe sonicating (90 W) for 45 sec.

Solutions of Ag^+ were prepared to the desired concentrations by dissolving $AgNO_3$ powder (Sigma Aldrich) in ultrapure Milli-Q water.

For the nitrification experiment, Ag treatments were mixed into 30 g of soil which was then separated into three 8 g replicates. In the sequencing experiment, Ag treatments were added to either 30 g or 5 g of soil depending on the target soil concentration. Silver treatments were added to soil either as a solution (Ag^+) or in suspension form (AgNP and Ag_2S-NP).

1.2. Characterisation of silver nanoparticles

The AgNP and Ag_2S-NP suspensions that were used in this experiment have been extensively characterised in previous studies using dynamic light scattering (DLS, Malvern Zetasizer), transmission electron microscopy (TEM, Phillips CM200 at 120 keV), X-ray diffraction analysis (XRD, PANanalytical X'Pert Pro) and UV – Vis absorption spectroscopy (200 – 600 nm) (Cary 5000 UV Vis NIR spectrophotometer) [2, 3]. The particle size distribution of AgNPs has also been investigated using disk

centrifuge analysis (CPS Instruments disc centrifuge 24000 UHR) [3]. Silver NPs and Ag_2S-NPs were uniformly dispersed and generally spherical with some rod-like particles (for TEM images of AgNPs and Ag_2S-NPs, see [3] and [2], respectively). The average hydrodynamic particle diameters (d_h) and zeta potentials (ζ) for AgNPs and Ag_2S-NPs were 44 nm and 152 nm, and -50 mV and -43 mV, respectively. The uniform dispersity of NP suspensions was evident from the close correlation between d_h and crystallite size (XRD, 41 nm) for AgNPs [3] and the relatively low polydispersity index (PdI, 0.21) recorded for Ag_2S-NPs [2]. These are the characteristics for 'pristine' AgNP and Ag_2S-NP suspensions; it is expected that in a real soil environment these properties may change.

1.3. Chemical analysis of silver concentrations in soil

Approximately 0.25 g of soil was digested in 50 ml Teflon® vessels with HCl (7.5 mL, 37%) and HNO_3 (2.5 mL, 70%), using a modified US EPA method 3051A [4]. Prior to microwave digestion, soils were open vessel digested at room temperature for 12 h. The temperature of vessels was then ramped using a CEM Mars Express system (1600 W) for 10 min to 175°C and maintained at 175°C for 45 min. The vessels were then cooled at room temperature and the digest solutions were diluted 2.5 times with HCl (10%), filtered (0.45 µm) and stored at 4°C until analysis. Blanks and a certified reference material (CRM [PACS-2]) were included in each digestion run. The Ag concentration of the digested CRM (1.24 ± 0.19 mg kg^{-1}) was in good agreement with the certified value (1.22 ± 0.14 mg kg^{-1}).

1.4. Potassium chloride extraction of soils and nitrate analysis

A 1 M solution of KCl was added to each subsample at a ratio 5:1 (soil to solution) and mixed (end-over-end) for 1 h to extract nitrate from soils. The samples were centrifuged (800 *g*, 5 min) and the supernatants filtered through a 0.45 µm mixed cellulose ester membrane filter (Millex®) and stored at -18°C until analysis. The nitrate (NO_3^-) concentrations in the liquid extracts were determined using flow-injection analysis (FIA) (Lachat QuikChem 8500 Series 2 FIA automated ion analyser system).

1.5. Quantitative polymerase chain reaction analysis of ammonia oxidising bacteria

Briefly, the PCR reaction contained 1x Biotaq SYBR green master mix (Biorad, Australia), 0.2 µM of forward and reverse primers, 5 µl of template DNA (1:10 dilution) in a total of 25 µL reaction. The PCR conditions were as follows; initial denaturation occurred at 95°C for 15 min, followed by 40 cycles of 95°C for 45 sec, 57°C for 60 sec, 72°C for 45 sec and a final extension of 72°C for 5 min. To confirm the specificity of amplified PCR products, all PCR reactions were followed by melting curve analysis and agarose gel electrophoresis. The melt curve conditions were 55°C to 95°C at a ramp rate of 0.5°C per 5 sec. Standard curves containing known copy numbers of the gene were generated using serial dilutions of linearised plasmids containing the *amoA* gene (from pure cultures of *Nitrosomonas* sp.); data were linear for 10^1–10^6 gene copies. Quantitative PCR was performed using a Maxpro 3000 qPCR machine (Stratagene, Australia) and data analysis was carried out using the software supplied.

1.6. Calculation of confidence intervals for curve parameters

Confidence intervals for *f*, and other parameters (including ECx values), were calculated as follows using a bootstrap technique:

$$f_{CI(95\%)} = f_{est} \pm (f_{SE} . t_{\alpha/2}) \qquad \text{(Equation 3)}$$

where $f_{CI(95\%)}$ = upper and lower 95% confidence intervals for parameter *f*; f_{est} = estimated value for *f* (calculated in R); f_{SE} = standard error of *f* (calculated in R); $t_{\alpha/2}$ = the critical value of the Student's *t* distribution at a 5% significance threshold (95% confidence interval) with nine degrees of freedom (2.262), where α/2 = 0.025.

References:

1. Doolette CL, McLaughlin MJ, Kirby JK, Batstone DJ, Harris HH, Ge H, et al. Transformation of PVP coated silver nanoparticles in a simulated wastewater treatment process and the effect on microbial communities. Chem Cent J. 2013;7:46-64.

2. Navarro DA, Kirby JK, McLaughlin MJ, Waddington L, Kookana RS. Remobilisation of silver and silver sulphide nanoparticles in soils. Environ Pollut. 2014;193(0):102-10.

3. Cornelis G, Doolette C, Thomas M, McLaughlin MJ, Kirby JK, Beak DG, et al. Retention and dissolution of engineered silver nanoparticles in natural soils. Soil Sci Soc Am J. 2012;76(3):891-902.

4. US-EPA. Method 3051A: Microwave assissted acid digestion of sediments, sludges, soils, and oils. Washington: Environmental Protection Agency, 1998.
